# The Impact of Bone Cement on Bone Healing in Revision Hip Arthroplasty for Periprosthetic Femur Fractures and Cortical Osteotomies

**DOI:** 10.7759/cureus.80399

**Published:** 2025-03-11

**Authors:** Amit Singh, Abhimanyu Singh, Dhaval Gotecha, Srikanth Gandavaram, Kuntal Patel, Deepak Herlekar

**Affiliations:** 1 Orthopaedics and Traumatology, Royal Lancaster Infirmary, Lancaster, GBR; 2 Orthopaedics and Trauma, Royal Lancaster Infirmary, Lancaster, GBR

**Keywords:** bone cement, bone healing, cortical osteotomy, periprosthetic fractures, revision tha

## Abstract

Background

It is widely acknowledged that bone cement may infiltrate the fracture site during the implantation of a cemented hip stem for a periprosthetic fracture, potentially leading to non-union. This study sought to examine this hypothesis through a radiological analysis of patients who underwent cemented femoral stem implantation to stabilize a periprosthetic femur fracture or after a cortical osteotomy for stem extraction.

Methodology

A retrospective study was conducted from 2015 to 2020 at a specialist center in the United Kingdom. Patients over 18 years old receiving cemented femoral stems for periprosthetic fractures or following cortical osteotomies were included. Bone healing was assessed through serial radiographs.

Results

The study included 25 patients with a mean age of 82.2 years and a female-to-male ratio of 16:9. Overall, 19 (76%) and 6 (24%) patients, respectively, received cemented femoral stems following a periprosthetic fracture revision or a cortical osteotomy used during revision. No bone grafts were used for any of the patients. Unfortunately, two patients were lost to follow-up, and five patients in the fracture group died before their fractures had united. The remaining 12 fractures healed in an average of 2.2 months, while all six cortical osteotomies healed in an average of 4.2 months.

Conclusions

Our research findings demonstrate the efficacy of employing a cemented femoral stem in revision hip arthroplasty scenarios involving periprosthetic femur fractures or cortical osteotomies, as it does not adversely affect bone healing.

## Introduction

Sir John Charnley first popularized the use of bone cement in total hip replacements in 1958 [1]. The primary component of bone cement is polymethyl methacrylate, which functions as a grout to secure the femoral stem against the bone [2]. According to the 20th UK National Joint Registry report, 3% of primary hip replacements required a first revision, with periprosthetic femur fractures being the third most common cause for these revisions [3].

The treatment approach for these injuries varies based on the fracture’s location and the stability of the implant. The increasing frequency of initial total hip arthroplasty (THA), along with an elderly population more prone to fall-related injuries, leads to a significant rise in revision surgeries [4]. Orthopedic surgeons will encounter an elevated incidence of periprosthetic fractures in older patients with osteoporotic bone, impaired mobility, diminished balance, and compromised cognitive function [5].

Geriatric individuals with periprosthetic fractures exhibit an elevated mortality risk, along with increased surgical and medical consequences [6]. Consequently, primary treatment objectives for geriatric patients significantly diverge from those of the younger age group. The goal of surgery is to achieve a quick recovery that allows for pain-free weight-bearing, all the while ensuring the integrity of the healing process for femoral fractures is maintained. [7]. Although periprosthetic femoral fractures may arise with any femoral component fixation technique, cement fixation has been associated with a reduced incidence of fracture [8,9]. The heightened risk of periprosthetic femoral fracture associated with cementless fixation is particularly significant in individuals with compromised bone quality, particularly among elderly and female patients [10,11]. A long-stem cemented prosthesis revision can facilitate early weight-bearing capability in these instances [12]. Nevertheless, cement use has been linked with the embolization of marrow components, leading to unfavourable effects on the cardiopulmonary system and possibly even early perioperative death [13,14].

Very few reports have been published on the outcomes of cemented stems in elderly patients undergoing revision THA. Hence, this study aimed to evaluate the impact of bone cement on bone healing when used in revision hip arthroplasty for periprosthetic femur fractures or following cortical osteotomies.

## Materials and methods

We identified two groups of patients for this study, namely, those with periprosthetic femur fractures and those requiring extraction of well-fixed femoral stems through cortical osteotomies. The study included all patients over the age of 18 years who were treated with cemented femoral stems during revision surgery for either of the two groups between 2015 and 2020. Patients under the age of 18 years, receiving a cementless revision, or with pathological fractures were excluded from the study.

An observational study was conducted on consecutive patients within a large NHS district hospital trust in the United Kingdom. This study was performed in line with the principles of the Declaration of Helsinki. Patient data were collected from electronic records, anonymized, and securely stored on a hospital computer accessible only to the research team. To confirm bone healing, the two revision arthroplasty surgeons reviewed serial radiographs at follow-ups, considering the bone healed if three cortices had united on anteroposterior and lateral radiographs. The patients’ operative notes were analyzed to identify any cement leakage across the fracture or cortical osteotomy site and to determine whether any bone graft substitutes were used, as these factors could impact bone healing.

Surgical procedure

All surgeries were conducted in a specialist arthroplasty medical center by two proficient revision arthroplasty surgeons utilizing a posterior approach with the patient in a lateral decubitus position. All revision surgeries were performed using Depuy C-stem AMT prosthesis. The hip joint was surgically dislocated; the acetabulum was evaluated for fixation and the polyethylene liner for wear. The posterior approach was extended distally, facilitating the removal of the stem and cement at the fracture/osteotomy site. Following further reaming of the diaphysis, the trial stem was introduced into the medullary canal, circumventing the distal fracture site by a minimum of two diaphyseal diameters to provide adequate stability [15]. The fracture pieces were realigned and secured with clamps, thereafter, undergoing trial reduction. Following the assessment of extremity length and implant stability, a minimum of two cerclage strips were affixed to the femur, and the long stem was positioned with the appropriate anteversion. Within the study group, the medullary canal was retrogradely filled with cement before stem insertion, and the fracture site was meticulously examined for cement leakage, followed by the removal of any extruded material. Cortical osteotomy was performed for the osteotomy group near the tip of the femoral stem as described previously in our case series [16].

Postoperative protocol

All patients received prophylactic antibiotics intravenously for 24 hours postoperatively. Venous thromboprophylaxis was administered for five weeks. Postoperative physiotherapy involved physical exercises aimed at muscle strengthening and hip mobilization. Mobilization commenced on the first postoperative day using a walker. Patients were permitted to bear weight as tolerated. Clinical union was also assessed at the time of follow-up visits and documented in patient notes; however, as a routine, radiographs were serially done. Clinical union before the radiological union was concluded if the patients were able to bear weight and were non-tender at the fracture site. Both the index consultants, who are trained and experienced revision arthroplasty surgeons, reviewed the patients at follow-up.

Postoperative follow-up schedule

All patients were followed up at one, three, six, and 12 months postoperatively with serial radiographs to assess for union.

## Results

A total of 25 (100%) patients meeting the screening criteria were enrolled in the study. The mean age was noted to be 82.2 years, with 16 (64%) being females. The baseline patient characteristics are presented below in Table 1.

**Table 1:** Baseline patient characteristics (N = 25). ASA = American Society of Anesthesiologists

Parameter assessed	Calculated value
Mean age (years)	82.24 ± 11.14
Median age with range (years)	84
Number of males (%)	9 (36%)
Number of females (%)	16 (64%)
Number of patients with ASA Grade 2 (%)	3 (12%)
Number of patients with ASA Grade 3 (%)	22 (88%)

**Figure 1:**
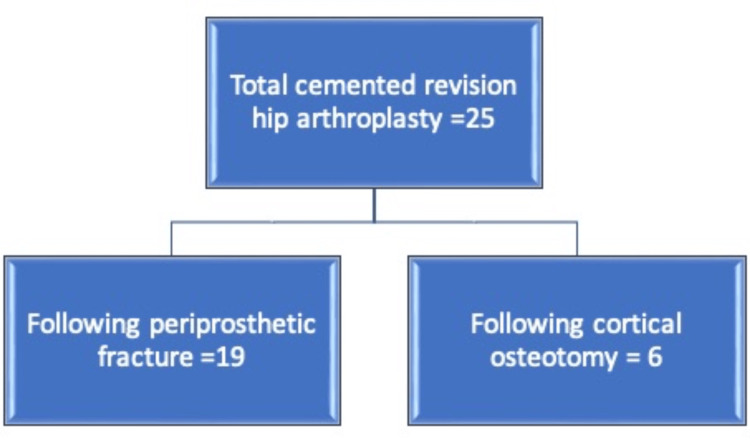
Patient distribution for subjects based on the indication of cemented revision hip arthroplasty.

Of the 25 (100%) patients in the study who underwent total cemented revision hip arthroplasty, 19 (76%) patients underwent the same following periprosthetic fracture, while six (24%) patients underwent cemented femoral stem implantation following a cortical osteotomy to extract femoral stems during revision surgery (Figure 1).

Cemented revision hip arthroplasty following a periprosthetic fracture

The mean age of the 19 (76%) patients in this subgroup was 81.46 years (SD = 10.58 years), with 13 being females (68.42%) while six (31.58%) were males. Of the 19 patients who underwent cemented revision hip arthroplasty following a periprosthetic femur, 17 (89.47%) were Vancouver type B2, one (5.26%) had B3, and another had type C femur fracture. Overall, seven (36.84%) of the patients in the subgroup also underwent open reduction internal fixation. One patient was lost to follow-up in the subgroup, and five (26.31%) patients expired before the fracture had united. The rest of the 13 (68.42%) patients had their fractures united in a mean period of 2.2 (±1.06) months. Table 2 highlights patients of the periprosthetic group with their demographic features, Vancouver fracture type, and surgical procedure.

**Table 2:** Details of the patients in the periprosthetic femur fracture group. ORIF = open reduction internal fixation; M = male; F = female

S. no.	Age	Sex	Vancouver Classification	Surgical fixation
1	84	F	B2	Femoral stem cemented revision plus ORIF
2	74	F	B2	Femoral stem cemented revision
3	87	F	B2	Femoral stem cemented revision
4	81	F	B2	Femoral stem cemented revision plus ORIF
5	89	M	B2	Femoral stem cemented revision plus ORIF
6	84	F	B2	Femoral stem cemented revision
7	79	F	C	Femoral stem cemented revision
8	87	M	B2	Femoral stem cemented revision
9	84	M	B2	Femoral stem cemented revision
10	80	M	B2	Femoral stem cemented revision
11	36	M	B2	Femoral stem cemented revision
12	94	F	B2	Femoral stem cemented revision plus ORIF
13	85	F	B3	Femoral stem cemented revision
14	84	F	B2	Femoral stem cemented revision plus ORIF
15	82	F	B2	Femoral stem cemented revision
16	76	F	B2	Femoral stem cemented revision
17	87	F	B2	Femoral stem cemented revision plus ORIF
18	85	F	B2	Femoral stem cemented revision
19	86	F	B2	Femoral stem cemented revision plus ORIF

Cemented revision hip arthroplasty following cortical osteotomy

The mean age of the six (24%) patients in this subgroup was 82.86 years (SD = 11.81 years), with an equal number of males and females. The indication for revision for five (83.33%) patients was aseptic loosening of the acetabular cup and infection for one (16.67%) patient. All patients were followed up successfully and had successful fracture unity (mean period to unity = 4.2 ± 2.3 months). The mean union time for patients with fracture and loosening was significantly different than that noted for the cortical osteotomy subgroup (2.2 ± 1.06 months versus 4.2 ± 2.3 months, p < 0.05 by unpaired t-test). Table 3 highlights patients of the cortical osteotomy group.

**Table 3:** Details of patients in the cortical osteotomy group. M = male; F = female

S. no.	Age	Sex	Indication for revision surgery
1	83	F	Aseptic loosening of the acetabular cup
2	88	F	Aseptic loosening of the acetabular cup
3	85	M	Aseptic loosening of the acetabular cup
4	92	F	Infection
5	70	M	Aseptic loosening of the acetabular cup
6	94	M	Aseptic loosening of the acetabular cup

Bone graft requirement and cement leak

None of the patients in the study received any bone grafts. No cement leak was observed intraoperatively across the fracture or osteotomy site for all 25 (100%) patients. One patient from the cemented revision group had a subsequent revision for dislocation.

## Discussion

Similar to primary THA, the stability of components in revision THA can be attained with or without the use of bone cement. The utilization of uncemented revision implants on the femoral side has experienced a growing trend in popularity. The results demonstrate exceptional long-term outcomes, especially in individuals with adequate femoral bone density [17]. Nevertheless, one possible drawback of uncemented revision stems is the limited weight-bearing permitted during the initial time after surgery to avoid excessive sinking of the stem, particularly when employing a transfemoral technique [18,19]. One technique to overcome this drawback of uncemented stems is by employing cemented femoral revision stems, which allow for rapid weight-bearing after surgery.

In our study, more than three-fourths of the patients indicated for revision THA had suffered from periprosthetic fractures after primary THA. Managing periprosthetic fractures of the femur following a THA can be a difficult task. The increasing number of initial THA procedures, coupled with a growing elderly population prone to falls, has resulted in a significant rise in the number of revision surgeries [4]. Older patients experiencing periprosthetic fractures are at an increased risk of mortality, along with a higher likelihood of encountering both surgical and medical complications [6]. As a result, the primary care objectives for older patients differ significantly from those for younger patients. The primary goal of surgery is to enable early, painless weight-bearing on the femur while ensuring proper fracture healing [7]. In our study, radiographic assessments showed that using a long stem cemented prosthesis for revision did not impede fracture healing. This was evident as the diaphyseal portion of the fracture healed in the cases followed up during the first year. Postoperative partial weight-bearing after hip revision may provide more challenges for older individuals with compromised balance [20]. Consequently, employing a long-cemented prosthesis can promote early weight-bearing in elderly patients, those with medical issues, and individuals who struggle to follow partial weight-bearing guidelines following hip revision surgery.

Aseptic loosening of an acetabular cup was the most common indication for cemented revision THA using a cortical osteotomy. Aseptic loosening may be due to insufficient initial fixation, gradual mechanical detachment, or biological detachment caused by osteolysis from particles around the implant [21]. These three elements are likely to have a combined effect in causing aseptic loosening in the clinical setting. The gradual weakening of the fixation, combined with deterioration from particles leading to the loss of biological attachment driven by osteolysis, creates a reinforcing cycle that ultimately contributes to progressive loosening. The meticulous and practical approach to revision surgery is crucial for preserving bone integrity, attaining favorable revision outcomes, maintaining hip function, and preventing complications.

Our study findings showed that bone cement for femoral stem implantation did not adversely impact bone healing in patients with either periprosthetic fracture or cortical osteotomy. Similar findings have been noted in other published studies. A recent study by Axenhus et al. compared the outcomes between a group of patients who underwent uncemented and cemented stem revisions in managing Vancouver B2 and B3 periprosthetic fractures. The cemented group noted lower dislocation and stem loosening instances, which were significant findings versus the uncemented group (p < 0.05). The clinical outcomes were similar between the cemented and the uncemented patient groups in the reference study [22]. Another study by Sponer et al. found that revision THA with long-stem cemented prosthesis provides early weight-bearing without any compromise on the healing of femoral fractures in elderly patients with osteoporosis, poor balance and mobility, and decreased cognitive competence [15].

One of the concerns for cemented prosthesis THA is the chance of cement extrusion, which can lead to non-union or refracture. In our study, none of the patients suffered from cement leaks during surgery. Corten et al. conducted a study on a group of 16 patients who had a healed diaphyseal part of a fracture after more than one year of follow-up [7]. In contrast, Springer et al. reported that three (9%) out of 34 patients who had undergone revision surgery had radiographic non-union after 68 months of follow-up [23]. To reduce the risk of non-union, it is crucial to handle the surrounding soft tissues with care to minimize disruption to the blood supply. Additionally, removing any extra cement and using either autogenous bone grafts or cortical strut allografts can also help ease this problem [23].

Dujardin et al. attempted to elucidate the paradoxical decrease in preference for cemented femoral stems in THA in their review [24]. Two objections are occasionally presented: the diminishing expertise in cemented stem implantation at training facilities, and a slightly increased duration of the surgical procedure. Nevertheless, it is important to note that the disparity in operational duration is just around 10 minutes. Additionally, factors such as the appeal of innovations and aggressive marketing by manufacturers may play a significant role. It is crucial to remain informed about these discussions and support them with rigorous empirical research. Otherwise, healthcare policies, both public and private, could be influenced by strong marketing tactics, leading to decisions made without thorough consideration. To prevent this, it is important to examine the reasons why surgeons do not rely on evidence-based advice [25].

Our study has given an overview of the utility and outcomes of cemented revision THA after periprosthetic fractures and cortical osteotomy. Nevertheless, this study has many constraints. The first issue is the loss of participants (24%) who did not continue to be involved in the study due to loss of follow-up or death. This was mainly because a significant number of patients were elderly. Additionally, the sample size was small (n = 25), and the overgeneralization of the results should be done with caution. We could not get more cases for analysis than what has been used for the study. The outcomes were evaluated radiographically, and other objective patient metrics were not assessed in the study. Nevertheless, the findings of the study are encouraging and further strengthen the confidence in cemented revision THA, irrespective of the cause. Further studies in a larger sample size with objective patient metrics or scores can help in the validation of our findings.

## Conclusions

Based on the results of our study and the current literature, we conclude that using bone cement for femoral stem implantation does not negatively affect bone healing in the presence of a periprosthetic fracture or cortical osteotomy. However, surgeons must still respect fracture biology by avoiding bone devascularization and removing any excess cement at the fracture or osteotomy site to promote optimal bone healing.
